# Multitask Attention-Based Neural Network for Intraoperative Hypotension Prediction

**DOI:** 10.3390/bioengineering10091026

**Published:** 2023-08-31

**Authors:** Meng Shi, Yu Zheng, Youzhen Wu, Quansheng Ren

**Affiliations:** 1 School of Electronics, Peking University, Beijing 100871, China; 2 College of Engineering, Peking University, Beijing 100871, China

**Keywords:** intraoperative hypotension, deep learning, multitask training, bio-signal prediction

## Abstract

Timely detection and response to Intraoperative Hypotension (IOH) during surgery is crucial to avoid severe postoperative complications. Although several methods have been proposed to predict IOH using machine learning, their performance still has space for improvement. In this paper, we propose a ResNet-BiLSTM model based on multitask training and attention mechanism for IOH prediction. We trained and tested our proposed model using bio-signal waveforms obtained from patient monitoring of non-cardiac surgery. We selected three models (WaveNet, CNN, and TCN) that process time-series data for comparison. The experimental results demonstrate that our proposed model has optimal MSE (43.83) and accuracy (0.9224) compared to other models, including WaveNet (51.52, 0.9087), CNN (318.52, 0.5861), and TCN (62.31, 0.9045), which suggests that our proposed model has better regression and classification performance. We conducted ablation experiments on the multitask and attention mechanisms, and the experimental results demonstrated that the multitask and attention mechanisms improved MSE and accuracy. The results demonstrate the effectiveness and superiority of our proposed model in predicting IOH.

## 1. Introduction

Intraoperative hypotension (IOH) is a common side effect during general anesthesia surgery. The more commonly used definition of this phenomenon is a mean arterial pressure of less than 65 mmHg during surgery. IOH is one of the risk factors for many postoperative complications, including renal failure [1,2,3], myocardial injury [4,5], organ dysfunction [2], stroke [6,7], and, in severe cases, even shock [8] and death [9,10,11]. Compared with other risk factors leading to these postoperative complications, IOH is relatively controllable and modifiable. Therefore, timely detection and treatment of IOH during surgery can help reduce the probability of these postoperative complications. Clinically, hypotension is usually treated with vasoactive drugs and fluid resuscitation.

During surgery, fully accurate prediction cannot be achieved even through intense manual monitoring, and it adds a considerable surgical burden to the medical staff. Machine learning and artificial intelligence techniques, on the other hand, are ideally suited to play a supporting role in the field of biomedicine [12,13,14,15], and can thus be considered for IOH prediction work. There have been a number of studies on the use of machine learning for IOH prediction, as exemplified in Table 1.

A prevalent theme in both biomedical research, such as IOH prediction, and broader time-series forecasting tasks is the intricate coupling of systematic feature engineering and kernel function optimizations to bolster model efficacy. Within the realm of machine learning, several models, including Random Forests, Gaussian Processes (GPs), and Relevance Vector Machines (RVMs), have shown their versatility in handling various tasks [16,17,18,19]. For instance, Lee et al. built a hypotension prediction model heavily reliant on feature engineering using random forests [20]. Diverging from the bio-signal prediction but remaining within the ambit of time-series analysis, Guan et al. proposed a Gaussian process model hinging on a fusion of both feature and kernel optimizations to improve long-term load forecasting [21]. Similarly, Qiu et al. delved into a multi-kernel relevance vector machine, meticulously extracting aging features for predicting the lifespan of lithium-ion batteries [22]. Although these methodologies have yielded commendable results, they rely on meticulous manual feature extraction and kernel optimization, which potentially stymies their broader applicability.

However, the trend in recent years leans towards more streamlined, end-to-end solutions that negate the need for manual feature crafting. Choe et al. opted for a fusion of Recurrent Neural Network (RNN) and Convolutional Neural Network (CNN), predicting hypotension over short intervals without the traditional feature extraction methods [23]. There is also a commercially available model which uses arterial pressure waveforms to calculate the hypotension prediction index (HPI) [24].

As mentioned above, most algorithms in the past have been limited to relying on a single data source, such as arterial pressure waveforms. However, in addition to the direct observation of arterial pressure to obtain a blood pressure value, blood pressure is also closely related to many physiological characteristics [25], including electrocardiogram (ECG), electroencephalogram (EEG), photoplethysmography (PPG), and carbon dioxide (CO_2_). Recently, Jo et al. predicted IOH using arterial blood pressure (ABP) and waveforms of ECG and EEG as well as a ResNet model [26]. Lee et al. created a multi-channel deep learning model based on 1D-CNN which predicts IOH using ABP, ECG, PPG, and CO_2_ [27].

**Table 1:** Representative studies on IOH prediction using machine learning in recent years.

Author	Model	Data Source	Innovation	Year
Hatib et al. [24]	Logistic Regression	IBP	Machine Learning Fine-Tuning	2018
Lee et al. [27]	CNN	IBP, ECG, PPG, CO_2_	Multi-Channel Data Source	2021
Choe et al. [23]	RNN and CNN	ABP	Weighted-Average of Deep-Learning Models	2021
Jo et al. [26]	ResNet	ABP, ECG, EEG	Multi-Channel Data Source	2022
Lee et al. [20]	Random Forests	IBP	Feature Extraction Model	2022

The work of Lee et al. inspires us to use multiple data sources to improve the prediction of our models, but there are some areas of their work that need to be improved and optimized. First, their selection of training data is overly stringent. In their data preprocessing method, only data with low blood pressure lasting for 60 s will be selected as an IOH sample, and only data with normal blood pressure lasting for 20 min will be selected as a normal sample. However, in real-world application scenarios, the patient’s ABP value will not be so ideal, and there is no guarantee that the data received by the model will meet such stringent requirements. In a more realistic scenario, the model would perform calculations and output predictions at short intervals, such as 10 s or so. We tried to construct a more realistic dataset, and when applying the 1D-CNN model constructed to this new dataset, the model performed much worse than the original. Second, although they considered multiple data sources, they did not further address studies that use multitask learning to predict IOH. In multitask learning, the same neural network has multiple different outputs, each corresponding to a different task, and it is able to mine features that correlate between different tasks to improve model performance. Our work unfolds based on these ideas.

In this paper, we adopt a more rationalized data preprocessing approach and propose a multichannel deep learning model for predicting IOH by multitask training and receiving multiple data sources, including ABP, ECG, PPG, and CO_2_. The model we use is ResNet-BiLSTM based on multitask learning and attention mechanism. The model directly outputs the predicted value of mean arterial pressure (MAP), and transforms the output into a classification task result by determining whether it is less than 65 mmHg. We chose to predict the blood pressure after 2 min because most vasoactive drugs take effect within 30 s of administration [28], and a prediction 2 min in advance is sufficiently long to allow the physician to take countermeasures. In comparison with three machine learning models dealing with time-series data, our model has sufficiently superior performance in regression as well as classification. Thus, our study provides an effective new strategy for predicting IOH and even for processing the physiologic signals of time-series. 

**Figure 1:**
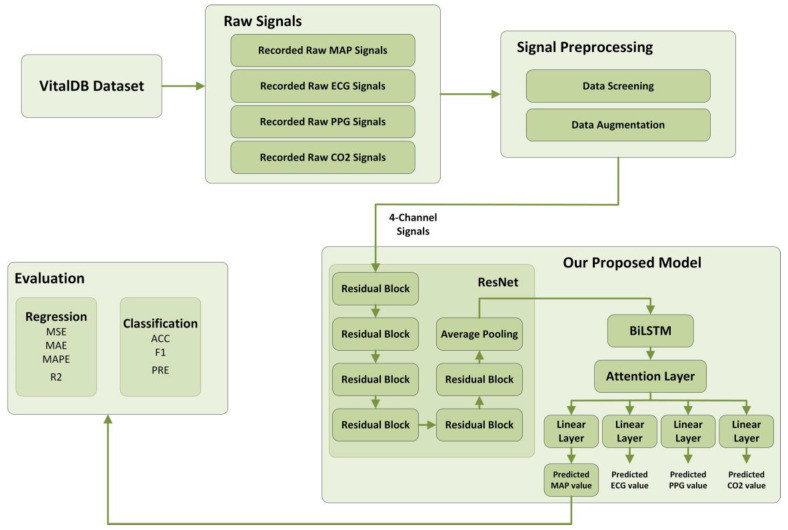
Our workflow.

The design of our study is illustrated in Figure 1. The rest of this paper is organized as follows: Section 2 describes the data and preprocessing methods we used as well as the structure of our multitask attention model; Section 3 reports the experimental results, including the comparison and ablation experiments; and Section 4 discusses our model in more depth, including its advantages, implications, etc. The conclusions are given in Section 5.

## 2. Materials and Methods

### 2.1. Dataset

The dataset used in this study is the open database VitalDB [29], which is specialized for machine learning studies related to vital signs. The dataset was collected by Seoul National University Hospital and contains data on multiple vital sign parameters of 6388 patients who underwent non-cardiac surgery, covering 196 intraoperative monitoring parameters, 73 perioperative clinical parameters, and 34 time-series laboratory outcome parameters, totaling 486,451 waveform traces. Data were from from 4 signals were used in this study, namely, ABP, ECG, PPG, and CO_2_.

### 2.2. Signal Preprocessing

The raw waveform data was sampled at 100 Hz, and the duration of each sample was set to 30 s. Therefore, there were a total of 3000 data point moments per sample. 

To delineate individual cardiac cycles, a peak detection algorithm was applied to the raw waveform. The peak detection parameters, such as height, prominence, and distance, were specifically tailored for each signal type, ensuring accurate rhythm segmentation. Several criteria were established to ensure data quality:

- Cardiac cycles that were detected as too slow, too fast, or undetectable were flagged as invalid.
- Data segments containing abnormal values in any of the four channels (e.g., ABP > 160 mmHg, ABP < 20 mmHg, ECG < −1 mV, ECG > 1 mV) were also excluded.
- Only segments maintaining a consistent rhythm for at least 10 min were considered valid.

The label of the sample was obtained by calculating the average ABP of 5 cardiac cycles after 2 min of input data. Eventually, data from 1378 patients were obtained. The total number of samples initially obtained was 1,215,362, of which 122,643 were hypotensive samples.

In the dataset, hypotension samples constituted only about 10% of the total. Such an imbalance could potentially influence the training outcomes. To address this, we utilized oversampling as our chosen data augmentation strategy. Specifically, we replicated each hypotension sample eight times, thereby increasing the number of hypotension samples to a total of 1,103,787.

### 2.3. Evaluation Metrics

In this paper, we first analyzed the predicted MAP values from the model output using regression metrics. Next, we classified the output based on whether the predicted MAP value is less than 65 mmHg, and then analyzed it using classification metrics. As shown in Table 2, the regression metrics we used include Mean Squared Error (MSE), Mean Absolute Error (MAE), Mean Absolute Percentage Error (MAPE), and coefficient of determination (R2). The classification metrics we used include Accuracy (ACC), F1 score (F1), and Precision (PRE).

In Table 2, *n* is the number of samples, ${\hat{y}}_{i}$ is the predicted MAP, $y_{i}$ is the ground truth value, *TP* is the number of true positive samples, *TN* is the number of true negative samples, *FP* is the number of false positive samples, and *FN* is the number of false negative samples.

### 2.4. Deep Learning Model

The overall structure of our proposed Multitask ResNet-BiLSTM-Attention for IOH prediction consists of ResNet structures, a BiLSTM, and an Attention mechanism. Each of these modules is described below.

#### 2.4.1. ResNet

ResNet is a deep neural network structure, meaning residual network, and the main idea is to build the network through residual connections to solve the problem of gradient vanishing and gradient explosion [30]. In traditional CNNs, simply stacking the network depth brings about the gradient vanishing, gradient explosion, and degradation problems. The degradation problem refers to the fact that as the network depth increases, the accuracy reaches saturation, and continuing to increase the network depth leads to a rapid decrease in accuracy. ResNet avoids the problem of gradient vanishing in deeper networks by introducing residual connectivity, which preserves the information from the previous layer. In residual learning, the function to be fitted is changed from H(x) to F(x) = H(x) − x, where x is called the constant transform and F(x) is called the residual function, making the network easier to optimize.

**Figure 2:**
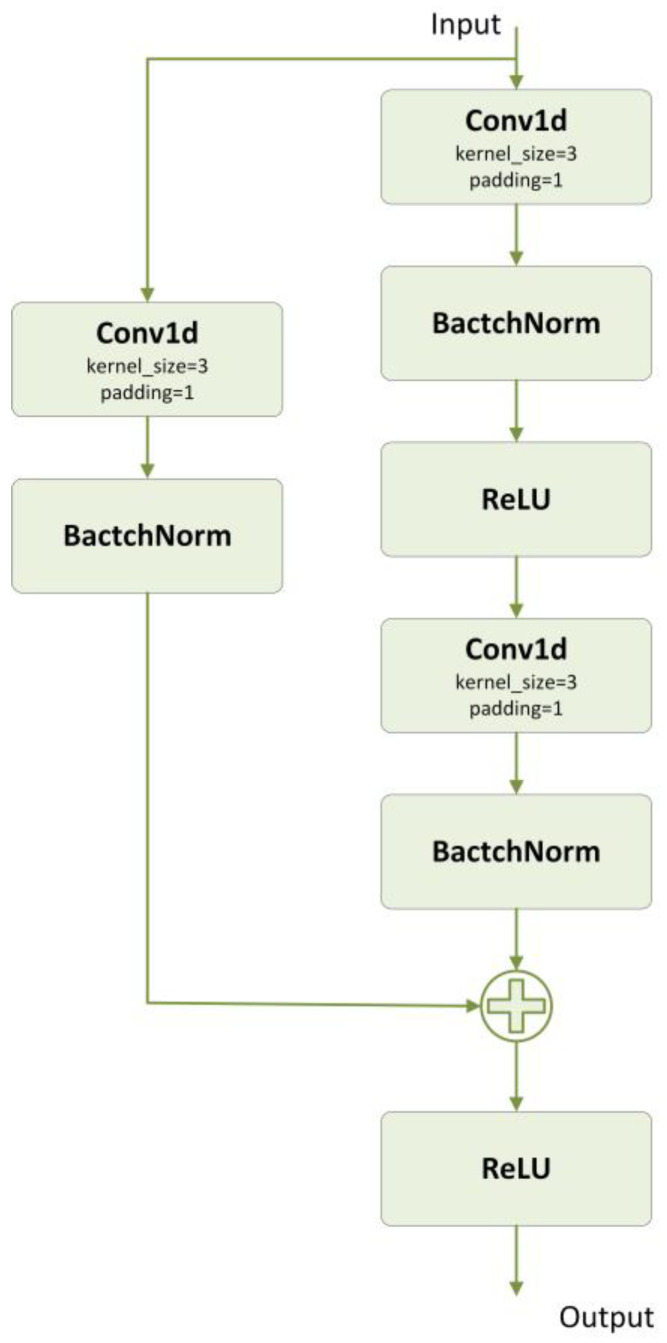
The structure of a residual block which we constructed.

A residual block consists of a convolutional layer, a batch normalization layer, an activation function (ReLU), and residual feedback. Figure 2 shows the structure of the residual block we have constructed.

#### 2.4.2. BiLSTM

BiLSTM [31] consists of two unidirectional and opposite LSTMs that process sequences from two directions. The outputs of the two LSTMs will be spliced after processing is complete. In forward LSTM, the input sequences are processed in left to right order, while in reverse LSTM, they are processed in right to left order. Forward and reverse LSTM learn the forward and backward feature representations of the sequence data in the time dimension, respectively. Compared to unidirectional LSTM, BiLSTM takes into account both past and future information, and thus understands the features of sequence data more comprehensively.

#### 2.4.3. Attention

**Figure 3:**
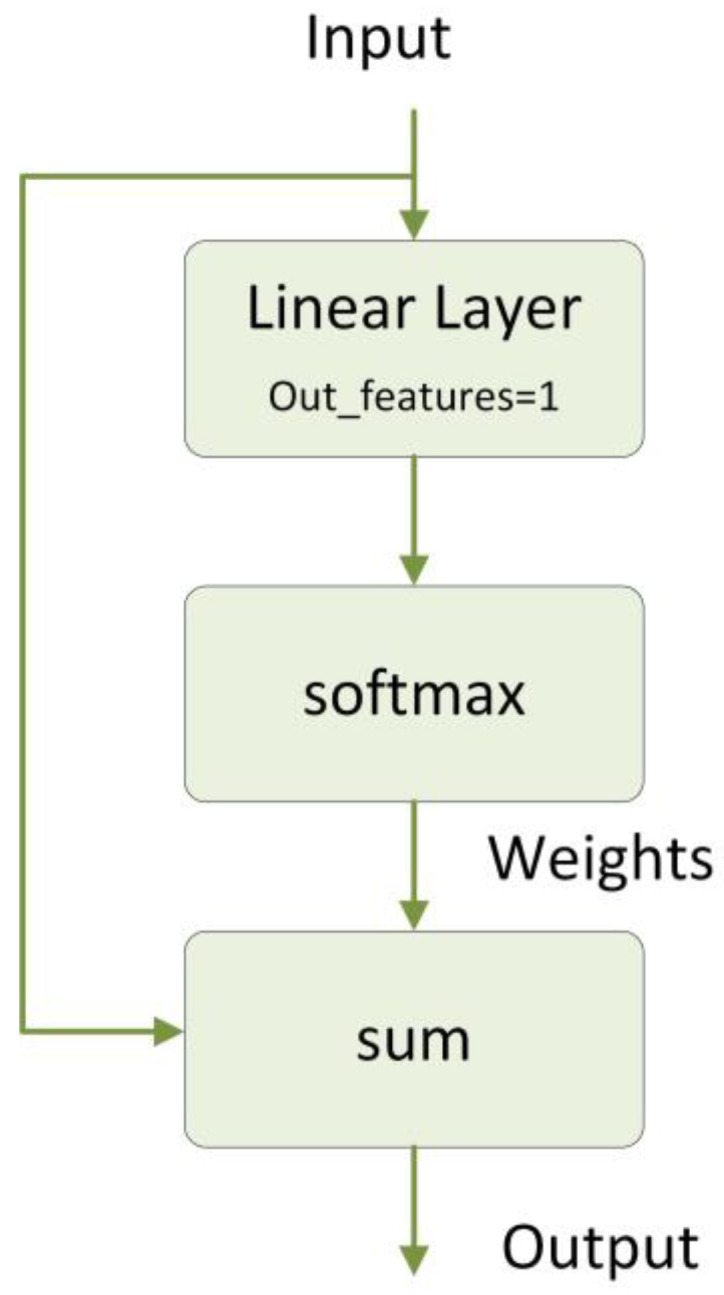
The structure of an attention layer we constructed.

The simplified version of the Attention module we built is shown in Figure 3. The input dimension is B × C × L, where B represents the batch size, C represents the number of channels, and L represents the length of data. After the fully connected layer as well as softmax outputs the data with dimension B × C × 1, which represents the attention weights of each time step, the input x is weighted and summed using the attention weights, and the final output dimension is B × L. Calculating the attention weights for weighting makes the important time steps receive more weights. The summation aggregates the information from different time steps and assigns more attention to the important time steps by weighting, resulting in a more meaningful and focused output.

#### 2.4.4. Multitask Learning

Multitask Learning [32] is a research direction in machine learning whose main goal is to improve the learning of each task by training multiple related tasks simultaneously. Compared with traditional single-task learning methods, Multitask Learning can more effectively exploit the correlation between multiple tasks, share representations and knowledge, and thus improve generalization ability and learning speed. It is also better able to handle data-scarcity situations because by sharing information, multitask models can draw on data from other tasks to improve task-specific performance.

A typical framework for multitask learning is a neural network, in which different tasks share the underlying network structure and have their own specific task heads. This structure allows the underlying feature representation to be shared between tasks, while the respective task heads are optimized specifically for each task.

#### 2.4.5. Our Proposed Model

**Figure 4:**
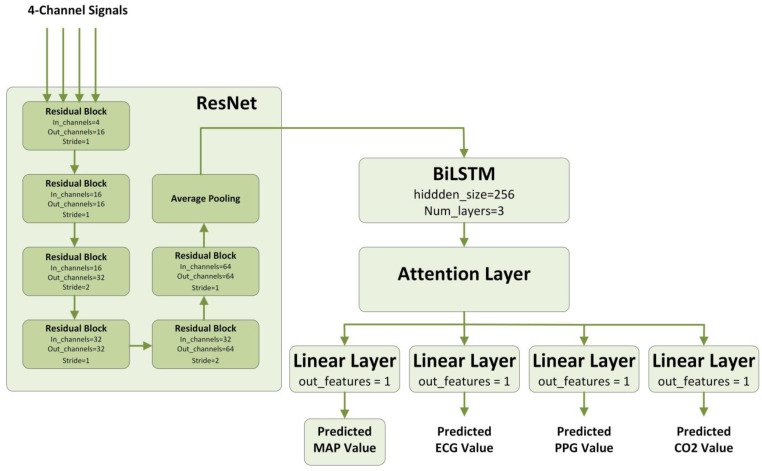
The structure of our proposed model.

Our proposed model consists of modules mentioned above and its architecture is shown in Figure 4. It includes a series of residual blocks, a BiLSTM, an Attention layer, and four fully connected layers to obtain the final outputs. The dimension of the input data is B × 4 × 3000, where B represents batch size, 4 represents the number of features, and 3000 represents the number of time steps. After a series of residual blocks as well as a pooling layer, the dimension of the data becomes B × 128 × 64. By stacking multiple residual blocks, we are able to construct deep neural networks and effectively solve the problems of gradient vanishing and gradient exploding, thus providing more powerful modeling capabilities for our task. Then, through the BiLSTM as well as the Attention layer, the information of each time step of the time-series is learned and integrated, and the outputs with dimensions of B × 128 × 512 and B × 512 are obtained, respectively. Finally, the data are passed into four fully connected layers, each of which is used to predict the value of a single physiological feature, respectively, and has an output dimension of B × 1.

Our choice of this hybrid architecture stems from the inherent challenges of IOH prediction. The fusion of ResNet and BiLSTM ensures the capture of intricate features and temporal dependencies, respectively, while the Attention mechanism allocates importance to the most pivotal data segments. Furthermore, the multitask learning approach augments the model’s generalization capabilities and enriches the extracted features, thus solidifying the architecture’s suitability for the intricate task of IOH prediction.

### 2.5. Machine Learning Models for Comparison

Three machine learning models used to analyze time-series data are selected for comparison in our study, including CNN, WaveNet, and TCN, to validate the performance of our proposed model.

#### 2.5.1. CNN

CNN [33], namely, Convolutional Neural Network, is a deep learning model specialized for processing grid-like data, such as images and audio. CNN contains a convolutional layer, a pooling layer, and, usually, a fully connected layer. It achieves efficient processing and feature extraction of data, such as images, by capturing local features in the input data through convolutional operations and reducing the data dimensionality through pooling layers. In this paper, we directly choose the 1D-CNN model built by Lee et al. in their paper for comparison.

#### 2.5.2. WaveNet

WaveNet [34] is designed to generate speech and audio data, and its main features are the efficient generation process and the high quality of the generated samples. WaveNet uses a structure of stacked convolutional layers which predicts the output at the current moment by considering inputs from multiple past moments. In this paper, we use a 10-layer WaveNet model.

#### 2.5.3. TCN

Temporal Convolutional Network (TCN) [35] is a model proposed in 2016 to solve time series forecasting. It is based on the CNN model with two improvements: the use of causal convolution to apply to the sequence model and the application of dilated convolution and residual blocks to memorize the history to achieve the effect of grasping the long time-dependent information.

## 3. Results

### 3.1. Experimental Settings

#### 3.1.1. Dataset Split

**Figure 5:**
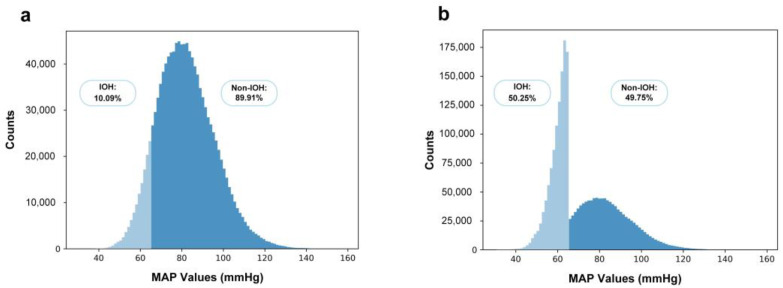
The distribution of data before and after data augmentation, where samples with MAP ≤ 65 mmHg are labeled as IOH samples. (**a**) The distribution of data before data augmentation, where 10.09% of the data are IOH samples. (**b**) The distribution of data after data augmentation, which is used in our study, where 50.25% of the data are IOH samples.

In our experiments, we randomly disrupted the data at the patient level, ensuring patient-wise consistency, and we subsequently divided the dataset according to the ratio of training set, validation set, and test set as 7:1:2. The data distribution before and after data augmentation is shown in Figure 5, where 50.25% of the data are IOH samples after data augmentation.

Each sample of the dataset contains time-series of four physiological indicators with 3000 data points each, corresponding to 30 s, and the dimensionality of the input data is 4 × 3000. The label of each sample of the dataset contains predictions for the four physiological indicators with the dimensionality of 4 × 1.

#### 3.1.2. Model Implementation and Training Details

Our proposed model and CNN, WaveNet, and TCN models were implemented using PyTorch [36]. The choice of hyperparameters was empirically determined based on a series of preliminary experiments and adjustments to optimize the model’s performance. The Adam optimizer was adopted for its observed advantages in convergence rate and stability during preliminary testing. A batch size of 128 achieved a balance between efficiency and generalization. The learning rate of 0.0001 was selected for consistent convergence, and the models were trained for 50 epochs.

#### 3.1.3. Hardware Configuration

All computational tasks were executed on an NVIDIA Tesla V100 (32 G) GPU, sourced from NVIDIA, Santa Clara, California, USA. To manage the expansive dataset and to prevent I/O bottlenecks, a memory allocation of 384 GB was utilized.

### 3.2. Experimental Results

#### 3.2.1. Comparison Experiments

Table 3 shows the results of the comparison between our proposed model and the three traditional models. In terms of regression performance, our model has the smallest MSE, MAE, and MAPE values (MSE: 43.83, MAE: 4.46, MAPE: 0.0545), and the largest R2 value (R2: 0.7731) compared to the conventional models (MSE ≥ 51.52, MAE ≥ 4.86, MAPE ≥ 0.0589, R2 ≤ 0.7502), which indicates that our model predicts MAP values with minor error and higher accuracy. In terms of classification performance, our model had the largest ACC, F1, and PRE values (ACC: 0.9224, F1: 0.6985, PRE: 0.6117) compared to the conventional models (ACC ≤ 0.9087, F1 ≤ 0.6625, PRE ≤ 0.5355), which suggests that our model has a better ability to differentiate between IOH and non-IOH samples.

**Figure 6:**
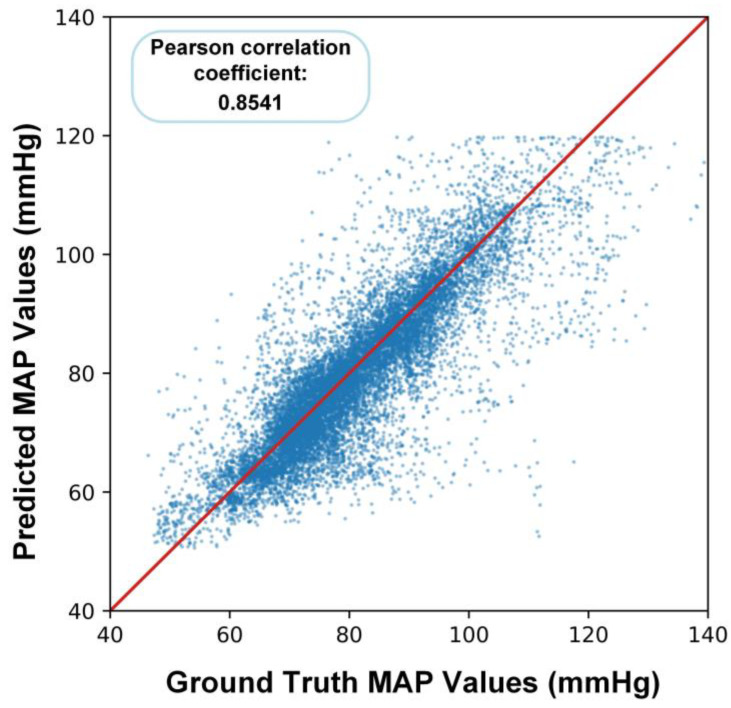
The scatter plot between a portion of the reference values and the corresponding model predictions of MAP. The red line represents the fitted curve in the best case, and the dots on the line represent the ground truth MAP values and the predicted MAP values being equal.

Figure 6 shows a scatter plot between a portion of the reference values and the corresponding model predictions of MAP. Their Pearson correlation coefficient is 0.8541, indicating a significant correlation between the predicted MAP and the reference values.

#### 3.2.2. Ablation Experiments

In this study, two improvements were made based on the ResNet-BiLSTM model: the model was trained using multitask method instead of single-task method, and the attention mechanism was inserted into the output module to enhance the model’s ability to focus on information at key time points. In order to verify the effectiveness of these two improvements, we conducted ablation experiments on these two improvements separately, producing a total of four sets of comparison results, as shown in Table 4.

It can be seen from the result that without adding multitask and attention mechanism, the MSE of the model is 50.67 and ACC is 0.9018. After adding one improvement, the MSE and ACC of the model are both improved, and only multitask makes the MSE decrease by 5.11 and the ACC increase by 0.0167, whilst adding only attention makes the MSE decrease by 2.40 and the ACC increase by 0.0160. It can be seen that multitask has a greater effect on the model’s improvement. Finally, the MSE and the ACC of the model were improved even more by adding both two improvements, with the MSE reduced by 6.84 and the ACC increased by 0.0206.

## 4. Discussion

In the landscape of models available for IOH prediction, our proposed model stands out with its unique hybrid architecture, multitask training, and attention mechanism.

Hybrid Architecture (ResNet-BiLSTM): The integration of ResNet and BiLSTM addresses specific challenges presented by the IOH prediction task. While ResNet offers the ability to mitigate the vanishing gradient problem by its shortcut connections, BiLSTM captures the forward and backward dependencies in time-series data. The fusion of these architectures results in better representation and understanding of bio-signal waveforms.

Multitask Training: Our decision to adopt multitask training is rooted in its capability to harness potential relationships between different yet related prediction tasks. Training the model on multiple tasks simultaneously not only improves its generalization but also facilitates extraction of a diverse array of features from the input data, enhancing the overall prediction performance.

Attention Mechanism: IOH prediction necessitates a model that can discern the critical segments in bio-signal waveforms. Our attention mechanism serves this purpose by allocating more weight to the informative segments of the data, ensuring the model focuses on the most pertinent parts for prediction.

Compared to the work of Lee et al., we constructed a hypotension dataset that is more suitable for real application scenarios. Experiments demonstrate that the model constructed by Lee et al. does not show better performance on our dataset, while the model we built is more suitable for real application scenarios. The data from the aforementioned comparison experiments show that with the addition of multitask and attention mechanisms, our model is an effective predictor of IOH with better prediction of MAP values as well as the ability to differentiate between IOH relative to conventional models. Furthermore, our model function is not limited to predicting blood pressure signals. As our model can effectively extract relevant features from biological signal waveforms, it is suitable for the prediction of more types of biological signals.

Integrating multitask training as well as attention mechanisms with time-series data, especially in the medical domain, is a notable innovation. The data from the aforementioned ablation experiments have shown the effectiveness of these two mechanisms, which provide unprecedented accuracy and reliability for predicting IOH in surgery, bringing practical, tangible value to the medical field.

We set the model as a regression task, but also obtained results for the classification task based on binarization judgments of the model’s output. However, there is still no standardized criteria for hypotension in clinical applications. Blood pressure varies from individual to individual, and the ability of individual organs to tolerate hypotension also varies [37]. Therefore, we believe that the results of the regression task are more valuable for reference and practical application.

Our model still has some challenges and limitations. First, although the deep learning model can provide highly accurate prediction results, it is difficult to provide an intuitive understanding of the decision-making process. This decision analysis is extremely important for the biomedical field, which determines the degree of acceptance of the prediction results for the medical staff. Another challenge lies in the large differentiation among different patients, where MAP data from different cases may vary or even contradict each other. The model’s learning of one patient case will better predict the MAP of this patient, but may not necessarily have the same accuracy to predict the MAP of other patients. How to improve the generalization ability of the model when encountering different patient cases is an issue that deserves further research.

## 5. Conclusions

In this paper, we propose the intraoperative hypotension prediction model based on a multitask ResNet-BiLSTM-attention model. The model simultaneously receives four physiologic time-series signals, ABP, ECG, PPG, and CO_2_, and outputs the prediction of the values of these four physiologic indices after 2 min. We performed comparative experiments between our proposed model and three conventional models used for time-series signal analysis, which showed that our proposed model outperformed these conventional models in regression and classification. We also conducted ablation experiments to verify the effectiveness of the multitask and attention mechanisms in this work. The experimental results demonstrate that our proposed model is an effective means for predicting IOH. The versatility of our proposed solution does not confine it to IOH prediction alone; its design potentially paves the way for forecasting a broader range of physiological signals in other medical applications.

## Figures and Tables

**Table 2:** Regression and classification metrics used in our work.

Metrics	Formula	Description
MSE (Regression)	$\frac{1}{n}\sum_{i=1}^{n}{\left({\hat{y}}_{i}-y_{i}\right)}^{2}$	Mean Squared Error
MAE (Regression)	$\frac{1}{n}\sum_{i=1}^{n}\left|{\hat{y}}_{i}-y_{i}\right|$	Mean Absolute Error
MAPE (Regression)	$\frac{100\%}{n}\sum_{i=1}^{n}\left|\frac{{\hat{y}}_{i}-y_{i}}{y_{i}}\right|$	Mean Absolute Percentage Error
R2 (Regression)	$1-\frac{\sum_{i=1}^{n}{\left({\hat{y}}_{i}-y_{i}\right)}^{2}}{\sum_{i=1}^{n}{\left({\bar{y}}_{i}-y_{i}\right)}^{2}}$	Coefficient of Determination
ACC (Classification)	$\frac{TP+TN}{TP+TN+FP+FN}$	Accuracy
F1 (Classification)	$\frac{2\times TP}{2\times TP+FP+FN}$	F1 Score
PRE(Classification)	$\frac{TP}{TP+FP}$	Precision

**Table 3:** The regression and classification results of our proposed model and three conventional models.

Metrics	WaveNet	CNN	TCN	Our Proposed Model
MSE	51.52	318.52	62.31	43.83
MAE	4.86	16.49	5.74	4.46
MAPE	0.0589	0.2010	0.0678	0.0545
R2	0.7502	−0.6732	0.6611	0.7731
ACC	0.9087	0.5861	0.9045	0.9224
F1	0.6625	0.3627	0.6129	0.6985
PRE	0.5355	0.2219	0.4678	0.6117

**Table 4:** The regression and classification results in the ablation experiments.

Metrics	ResNet-BiLSTM	ResNet-BiLSTM + Attention	ResNet-BiLSTM + Multitask	ResNet-BiLSTM + Multitask + Attention
MSE	50.67	48.27	45.56	43.83
MAE	4.91	4.76	4.74	4.46
MAPE	0.0604	0.0575	0.0579	0.0545
R2	0.7234	0.7599	0.7578	0.7731
ACC	0.9018	0.9178	0.9185	0.9224
F1	0.6014	0.6418	0.6484	0.6985
PRE	0.4820	0.5319	0.5457	0.6117

## Data Availability

The code of our proposed model will be available after acceptance, and the dataset of this study is available from the corresponding author upon reasonable request.
